# Supplementary material for: Refining perovskite structures to pair distribution function data using collective Glazer modes as a basis
Source: IUCrJ. 2022 Sep 1;9(Pt 5):705–12. doi: 10.1107/S2052252522007680 (PMC9438497; doi:10.1107/S2052252522007680)
Supplement: Supplementary file 1 [file m-09-00705-sup1.pdf]

IUCrJ

Volume 9 (2022)

Supporting information for article:

Refining perovskite structures to pair distribution function data using collective Glazer modes as a basis

Sandra Helen Skjærvø, Martin A. Karlsen, Riccardo Comin and Simon J. L. Billinge

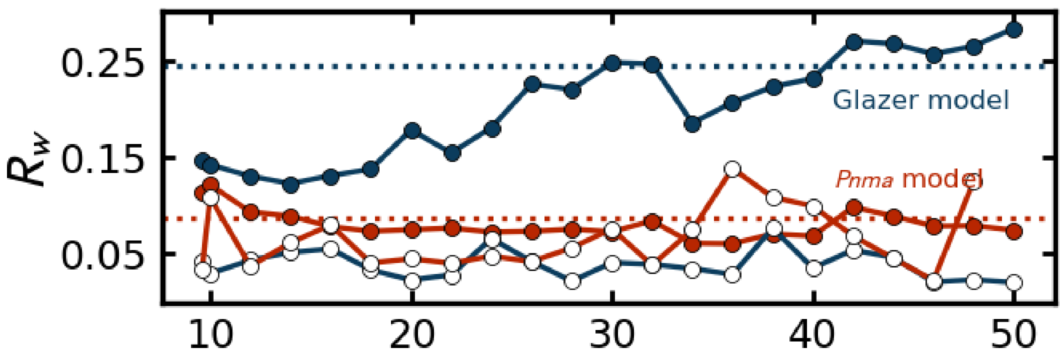

Figure S1: Comparison of the fit residual R_w from boxcar fits with the space group model (blue) and the Glazer model (red). The closed circles are fits of measured PDF data of $CaTiO_3$ at 200 K while the open circles are fits to simulated PDF of $CaTiO_3$. The simulated data were calculated from a constructed 2x2x2 unit cell with tilt system $\alpha^+ \beta^- \beta^-$, where tilt angles $\alpha = 9^\circ$ and $\beta = 10^\circ$. The Ca displacements and the isotropic thermal displacement parameters (U_{iso}) for each element were chosen according to fits with the space group model of the measured $CaTiO_3$ at 200 K.
